# Fungal ventricular assist device infections occur in colonized patients and are associated with high mortality rate

**DOI:** 10.1186/cc12024

**Published:** 2013-03-19

**Authors:** P Gaudard, J Eliet, N Zeroual, G Culas, P Colson

**Affiliations:** 1 CHRU Montpellier, France

## Introduction

Infection is a common complication of the ventricular assist device (VAD) and is associated with poor outcome especially with fungi [1]. The relationship between colonization and invasive fungal infection (IFI) in severely ill ICU patients with a VAD support is not described. This study analyzes the incidence and outcome of fungal infection and colonization in VAD patients in bridge to transplantation or in destination therapy.

## Methods

We conducted a retrospective review of all VAD implantations in our surgical ICU between 2007 and 2012. The incidence of fungal colonization, antifungal prophylaxis, bacterial sepsis and the mortality of IFI versus no IFI patients were compared.

## Results

In the study period, 34 patients with severe heart failure or cardiogenic shock were selected for a VAD implantation (nine in destination therapy). The overall mortality rate was 50% during mechanical assistance. Confirmed (*n *= 8) and highly suspected (*n *= 2) IFI occurred during the ICU stay in 29% of patients who were treated with echinocandins, voriconazole and/or liposomal amphotericin B. The isolated fungi were: six *Candida albicans*, two parapsilosis, one glabrata and one invasive pulmonary aspergillosis. Antifungal prophylaxis with fluconazole was administered to 18% of patients at mean for 5 days mainly in the more recent implantations. In the no IFI population, 54% (*n *= 13) had a systemic or VAD bacterial sepsis with a mortality rate about 54%. The mortality without any sepsis was reduced to 18%. Fungal colonization was significantly more present (90% vs. 50%) before IFI in VAD patients. The mortality rate was dramatically higher with IFI (80% vs. 38%) in accordance with the literature [1]. See Table 1.

**Table 1:** Fungal colonization (FC), infection and outcome during VAD support

	*n*	FC	Mortality
No IFI	24	12	9
IFI	10	9	8
*P *value		0.029	0.024

## Conclusion

In our center, we observed a high incidence of IFI in ICU patients with VAD that was associated with a mortality rate of 80%. Screening of fungal colonization appears to be very important during the ICU stay for VAD patients. Trials are needed for investigating the use, the drug choice and the timing of antifungal prophylaxis for such high-risk patients.
